# Pirfenidone Alleviates Choroidal Neovascular Fibrosis through TGF-*β*/Smad Signaling Pathway

**DOI:** 10.1155/2021/8846708

**Published:** 2021-02-10

**Authors:** Chuang Gao, Xin Cao, Lili Huang, Yueqi Bao, Tao Li, Yue Di, Liucheng Wu, Yu Song

**Affiliations:** ^1^Department of Ophthalmology, Affiliated Hospital 2 of Nantong University, Nantong, Jiangsu 226001, China; ^2^Department of Ophthalmology, Tongzhou People's Hospital Affiliated to Nantong University, Nantong, Jiangsu 226300, China; ^3^Department of Ophthalmology, Wuxi Children's Hospital, Wuxi 214002, China; ^4^Laboratory Animal Center of Nantong University, Nantong, Jiangsu 226001, China

## Abstract

*Background.* Transforming growth factor-*β* (TGF-*β*) plays a major role in CNV. However, the mechanism is unclear. This study investigates the effect of Pirfenidone (PFD) on TGF-*β*/Smad signaling pathway on the development of choroidal neovascular fibrosis in choroidal neovascularization (CNV) mouse model. C57BL/6J male mice (aged from 6 to 8 weeks) received intravitreal injections of phosphate-buffered saline (PBS)/PFD solution on 14 days after laser injury. Mice were anesthetized by intraperitoneal injection of 4% pentobarbital (0.05 mg/g body weight). Optical Coherence Tomography (OCT), Fundus Fluorescein angiography (FFA), and hematoxylin-eosin (HE) were used to assess CNV formation. The fibrosis area was monitored by staining the collagen type I (Col-I). Western blotting was used to analyze the expression of TGF-*β*2, Smad 2/3, phosphorylated Smad 2/3 (p-Smad 2/3), and *α*-smooth muscle actin (*α*-SMA). Terminal deoxynucleotidy1 transferase dUTP nick-end labelling (TUNEL) assay was performed on cryosections of mouse eyes to detect apoptosis. Our data showed PFD inhibited areas of fibrosis during day 21 to day 28. We also found that the levels of TGF-*β*2 protein expressions increasingly reached the peak till the 3rd week during the CNV development. The protein levels of Smad 2/3, p-Smad 2/3, and *α*-SMA also increased significantly in CNV mice, but this response was profoundly suppressed by the TGF-*β* inhibitor PFD. The results of this study suggest that TGF-*β*2 represents a target to prevent or treat choroidal neovascular fibrosis, and PFD may provide an alternative to traditional methods for Wet Age-related macular degeneration (wAMD) treatment.

## 1. Introduction

AMD has become the first cause of blindness in Western countries [1], which is one of the main causes of blindness in the world's elderly population; almost all cases of advanced AMD occur in people over the age of 60 [2]. It is classified as nonexudative or dry AMD and exudative or wAMD. wAMD is an advanced stage of AMD characterized by CNV which can cause bleeding, fluid accumulation, and fibrosis within the macula. Although the incidence of wAMD is only 15%–20%, the blindness rate is as high as 90% [3]. AMD is a fundus disease caused by multiple factors, and CNV formation is the main pathological change of wAMD [4]. CNV refers to the choroidal capillary entering the subretinal space through the Bruch membrane, causing retinal pigment epithelium (RPE) detachment, subretinal hemorrhage, and eventually permanent subretinal fibrovascular scar formation, leading to retinal tissue damage and central vision loss [5]. The current treatment of wAMD is the intravitreal injection of anti-VEGF drugs, but there are limitations, such as repeated injection, high cost, and poor compliance of patients [6, 7]. In addition, inhibition of VEGF alone is not enough to control the process of wAMD. It also has the potential to promote the development of subretinal fibrosis [8]. Therefore, alternative therapeutic approaches, which potentially attenuate subretinal fibrosis development, would fulfill an unmet medical demand in the treatment of wAMD.

TGF-*β* is a versatile fibrotic remodeling medium that can induce myofibroblast transformation, Smad activation, and extracellular matrix (ECM) generation [9]. TGF-*β* signals are transduced mainly by TGF-*β* receptor (T*β*R)-mediated Smad and non-Smad signaling. Activated TGF-*β* binds to TGF-*β* receptor 2 (TGF*β*BR2) and activates TGF-*β* receptor 1 (TGFBR1), resulting in phosphorylation of Smad 2 and Smad 3 [10]. p-Smad 2/3 and smad 4 form the Smad complex [11] and then transport to the nucleus to regulate the expression of genes such as collagen, fibronectin, and *α*-SMA [12], and finally form tissue fibrosis. Studies by Wang Xiaolei confirmed that the TGF-*β*plays has an important role in the occurrence and development of CNV by regulating proangiogenic factors [13]. TGF-*β*2 plays the main role in the homeostasis of the eye under physiological conditions. In animal models, Zhang et al. found that inhibiting the antagonism of TGF-*β* and COX-219 and then downregulating the expression of TGF-*β*2 can reduce subretinal fibrosis after CNV induction [14]. Therefore, multiple lines of evidence indicate the potential of TGF-*β*/Smad signaling as a therapeutic target in ocular angiogenic disorders marked by tissue fibrosis. Thus, we considered that inhibiting TGF-*β*2might be a novel strategy to attenuate the fibrosis of wAMD.

PFD is a new type of broad-spectrum antifibrotic complex [15]. Previous studies have demonstrated that PFD exerts antioxidant, anti-inflammatory, and antifibrotic activity. In vitro and animal experimental data show that PFD can delay or even reverse fibrosis and scar formation. Previous studies have shown that PFD can inhibit connective tissue growth factor (CTGF), platelet-derived growth factor, *α*-SMA, and TGF-*β* [16–18]. In the present study, a CNV mouse model simulating wAMD was established by laser photo coagulation in order to observe whether PFD can prevent the formation of choroidal neovascular fibrosis by inhibiting TGF-*β*2 and provide more experimental foundation and basis for finding more specific targets and safer interventions of wAMD.

## 2. Materials and Methods

### 2.1. Animals

C57BL/6J mice aged 6–8 weeks from Shanghai were maintained and bred at the Experimental Animal Center, Nantong University (Jiangsu, China). This study complies with ARVO's statement on the use of animals in ophthalmic and visual studies. Approval was obtained from the Animal Research Ethics Committee, Nantong University, in agreement with the Chinese National Standard. All the experimental steps were carried out according to the requirements of the animal welfare committee of Nantong University. [permit nos. SYXK (Su) 2017–0046 and SCXK (Su) 2019–0001].

### 2.2. Laser-Induced CNV and Drug Treatment

Anesthesia was induced in 100 mice through inhalation of isoflurane (induction: 5%, maintenance: 1%). After dilated pupils of compound tropicamide in both eyes, the corneas were anesthetized with 0.5% proparacaine hydrochloride eye drops. Laser photocoagulation (532 nm, 200 mW, 0.05s duration, 50 mm spot size) was performed with four laser burns in the 3, 6, 9, and 12 o'clock position of the posterior pole of the fundus with the distance of 2 disc diameters from the optic nerve head. No retinal hemorrhage and air bubbles are generated as a successful standard for laser photocoagulation. All mice were randomly divided into four groups based on the time following laser treatment (day 7, 14, 21, and 28).

### 2.3. OCT and FFA

Fundus examination of mice was performed with OCT under general anesthesia and dilated pupils. FFA images were taken 2–5 minutes after intraperitoneal injection of 0.3 ml of 2% sodium fluorescein to assess the development of CNV.

### 2.4. HE Staining

Eyes were enucleated and fixed for 4 hours at room temperature (RT) in 4% paraformaldehyde (PFA). They were washed in 0.1 M sodium phosphate buffer (pH 7.4) and sequentially cryoprotected in 20% and 30% sucrose. After the tissues were submerged, they were frozen and continuously sliced to a thickness of 5 *μ*m and stored at −20°C. HE staining was carried out using the standard protocol. Then, light microscopy was used to observe the tissue.

### 2.5. Intravitreal Injection

1 *μ*l of PFD or vehicle (PBS solution) was administered on day 14. PFD was injected into the mid‐vitreous cavity in the right eye, and the fellow eye was injected with PBS. Then erythromycin eye ointment was applied to prevent infection.

### 2.6. TUNEL Assay

Healthy mice were sacrificed one day after intravitreal injection of PFD or PBS solution. Apoptotic cells were detected by TUNEL assay. The assay was performed using the YF®488 TUNEL Assay Apoptosis Detection Kit (US EVERBRIGHT® INC). According to the manufacturer's protocol and was analyzed by fluorescence microscopy. The frozen sections were placed at room temperature for 20 minutes, dried and immersed in 4% paraformaldehyde solution, and fixed at room temperature for 30 minutes. Wash the sections with PBS infiltration twice for 5 min each. Slices were treated with proteinase K for 30 minutes at room temperature and washed in PBS. The sections were incubated with TdT enzyme at 37°C for 2 h, washed sample 3 times with a buffer of 0.1% Triton X-100 in PBS containing 5 mg/mL BSA for 5 min each. Retinal sections were counterstained with DAPI to reveal cell nuclei.

### 2.7. Analysis of Lesion Area in RPE/Choroid Flat Mounts

The areas of choroidal fibrotic tissue were measured on day 21 and day 28 after laser injury. The anterior segment and the neural retina were removed, and the eyecups were fixed in 4% paraformaldehyde for 1 h and then washed with cold immunocytochemical buffer (0.5% BSA, 0.2% Tween-20 and 0.1% Triton X-100) in PBS, and then collagen type I antibodies (1 : 100 dilution, Abcam, Cambridge, MA, USA) were added and incubated at 4°C overnight. After staining, the eyecups were flattened by four to five radial cuts from the edge to the equator and sealed with microscopic analysis.

### 2.8. Western Blot

To quantify protein levels, the RPE-choroid complexes of 5 mice were extracted at 7, 14, 21, and 28 days after the laser injury and placed into RIPA buffer with protease inhibitors and phosphatase inhibitors. After mechanical disruption, lysates were centrifuged at 13000 rpm for 10 minutes at 4°C. The supernatants were collected and preserved at −80°C. The proteins and a molecular weight marker were separated by 10% sodium dodecyl sulfate–polyacrylamide gel electrophoresis (SDS–PAGE) and transferred to a polyvinylidene difluoride (PVDF) membrane. The membrane was then incubated with primary antibodies for TGF-*β*2 (1 : 1000, ab36495, Abcam, UK), Smad2/3 (1 : 1000, catalogue number #5678), p-Smad 2/3 (1 : 1000, catalogue number #8828), and *α*-SMA (1 : 1000, ab32575, Abcam, UK). The antibodies were incubated in 5% skim milk or BSA overnight at 4°C and reacted with horseradish peroxidase (HRP)-conjugated secondary antibodies (1 : 2000, protein tech, USA) at room temperature for 2 h. Extensive washes in 0.05% Tween-20 in Tris-buffered saline (TBS) were followed by incubation with anti-GAPDH (1 : 2000, AT0002, CMCTAG, USA). The blots were then incubated with the chemofluorescent reagent enhanced chemiluminescence (Western Bright ECL, K-12043-D10, Advansta, USA) and exposed to X-ray film in the dark. The intensity of the GAPDH signal was used as endogenous control, and the band optical density was quantified using Image *J*.

### 2.9. Statistical Analysis

All experiments were repeated at least 3 times. All data were presented as mean ± standard deviation (SD). Values were performed using Student's t-test between the two groups. Three groups of differences were compared by one-way ANOVA, and the pairwise comparisons between groups were tested by LSD-t. Statistical analyses were conducted using SPSS 23.0. *P* < 0.05 was considered to be statistically significant.

## 3. Results

### 3.1. Establishment of the CNVmodel

**Figure 1:**
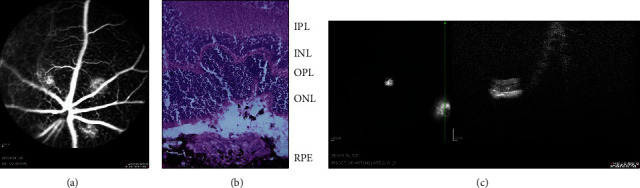
Changes in the mouse retina 1 week after laser photocoagulation. (a) FFA reveals strong fluorescent leakage at the fundus injury point one week after laser photocoagulation. (b) HE staining results show that Laser-induced CNV presented vascular plexus with wide blood vessel lumen which originated from the choroid and grew towards the bottom of the retina. (c) A subretinal spindle-shaped hyperreflective lesion represented the development of neovascularization. Scale bar: 50 *µ*m. RPE: retinal pigment epithelium; OS: outer segment; IS: inner segment; ONL: outer nuclear layer; OPL: outer plexiform layer; INL: inner nuclear layer; IPL: inner plexiform layer; GCL: ganglion cell layer.

One week after the laser injury, the lesion was easily distinguished from the surrounding retina on OCT and showed that the RPE/choroid capillary light bands were enhanced and the lower part was medium and high backscattering (Figure 1(c)), FFA showed obvious leakage of fluorescein (Figure 1(a)). Histological analysis of CNV lesions in HE stained retina showed damage to RPE, the outer nuclear layer, outer plexiform layer, and the inner core layer. Fibrovascular complexes (including collagen fibers, fibroblasts, endothelial cells, RPE cells, and neovascularization extend into the subretinal space (Figure 1(b))). This indicates that CNV formation occurred 1 week after laser injury, which suggests that the model is successful.

### 3.2. PFD Inhibits the Induction of Fibrosis Marker Proteins

**Figure 2:**
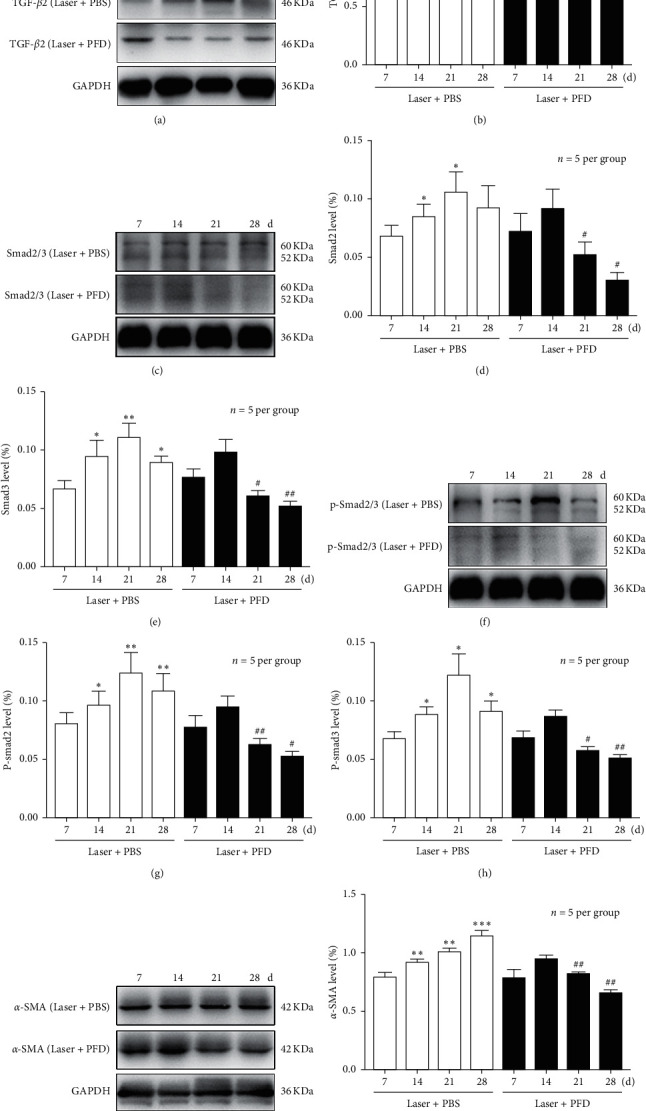
Intravitreal injection of PFD attenuates the formation of fibrosis. Representative images of collagen I (red) staining of the retinal pigment epithelium-choroid-sclera flat mounts obtained from the three groups. Scale bar: 200 *µ*m. Quantitative measurement of the fibrosis area. ^*∗∗*^*P* < 0.01 vs. Laser and Laser + PBS. #*P* < 0.05 vs. day 21.

**Figure 3:**
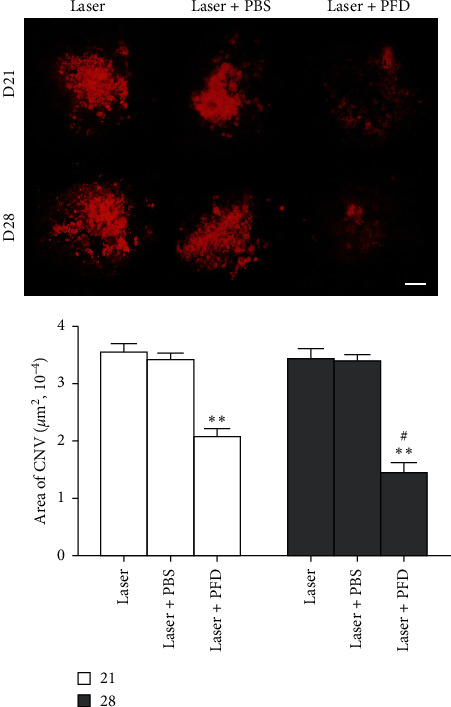
Intravitreal injection of PFD downregulates the expression of TGF-*β*2: Smad2/3: p-Smad2/3 and *α*-SMA. (A: C: F: I) Western blot showed the expression of TGF-*β*2: Smad2/3: p-Smad 2/3 and *α*-SMA in the retinal pigment epithelium-choroid-sclera complex after intravitreal injection of PFD or PBS. (B: D: E: G: H: J) Quantification graphs for TGF-*β*2: Smad 2/3: p-Smad 2/3 and *α*-SMA. Data of the relative protein level normalized to that of GAPDH are presented as the mean ± SD. ^*∗*^*P* < 0.05: ^*∗∗*^*P* < 0.01 vs. day 7. #*P* < 0.05: ##*P* < 0.01 vs. the Laser + PBS group.

To identify the effects and mechanisms of PFD in choroidal neovascular fibrosis, intravitreal injection of PFDand PBS were performed 14 days after laser treatment. As shown in Figure 2, the TGF-*β*2 protein expression levels in retina-choroid complex during the development of laser-induced CNV formation was determined by using Western blot. In the Laser + PBS group, the quantitative analysis results demonstrated that the protein expression levels of TGF-*β*2 increased significantly from 1 to 4 weeks and reached the peak at day 21 in the retina-choroid complex of mice after Laser-induced CNV formation (Figures 2(a) and 2(b)). PFD treatment could significantly suppress the increase of TGF-*β*2 protein expressions in RPE-choroid complex of mice. We next examined the effects of PFD treatment on the expression level of a protein marker of fibrosis. We found that the expression levels of *α*-SMA protein were significantly elevated and proportional to time in the PBS group. But this increase in protein expression was largely abrogated by PFD treatment (Figures 2(i) and 2(j)). Furthermore, to further investigate the effect of TGF-*β*2overexpression on collagen synthesis, the area of fibrosis was investigated by using RPE/choroidal flat mounts stained with Col-I (a marker of fibrosis) at 3–4 weeks following CNV induction. On day 21, the fibrotic area was 339.661 ± 16.649 *μ*m^2^ in the Laser group and 336.283 ± 18.581 *μ*m^2^ in the Laser + PBS group, compared to 205.618 ± 14.582 *μ*m^2^ in the Laser + PFD group (*P* < 0.001). On day 28, it was 344.327 ± 18.536 *μ*m^2^ in the Laser group and 339.949 ± 16.460 *μ*m^2^ in the Laser + PBS group, compared to 168.523 ± 20.892 *μ*m^2^ in the Laser + PFD group (*P* < 0.001) (Figure 3). These results suggested that PFD may reduce the expression of a-SMAand Col-Iby decreasing the expression of TGF-*β*2, thereby reducing the formation of choroidal neovascular fibrosis.

### 3.3. PFD Suppressed TGF-*β*/Smad Signaling Pathway

To further evaluate the effects of PFD, we examined whether the TGF-*β*/Smad signaling pathway was related to the choroidal neovascular fibrosis in mice and whether PFD could inhibit this pathway. The protein expression of Smad 2 and Smad 3, as well as the phosphorylation of Smad 2 (p-Smad 2) and Smad 3 (p-Smad 3), was examined (Figure 2(c) and 2(f)). Basing on the analysis of Western blot in the Laser + PBS group, the expressions of Smad 2/3 and p-Smad 2/3 were significantly increased at 1–4 weeks after Laser-induced CNV formation (Figures 2(d), 2(e), 2(g), and 2(h)). And besides, these increases in protein expression were largely abrogated by PFD treatment (*P* < 0.05). These results demonstrated that PFD suppressed TGF-*β*/Smad signaling pathway in mice with choroidal neovascular fibrosis.

### 3.4. Cytotoxicity of PFD in Retinal Cells

**Figure 4:**
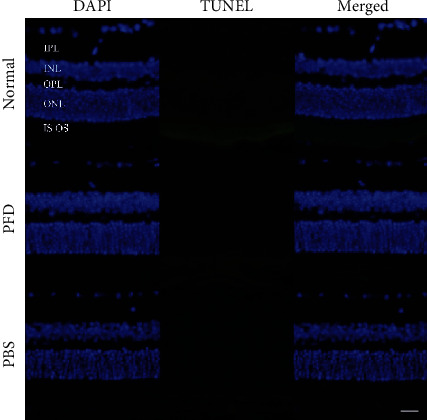
. Intravitreal injection of PFD does not cause apoptosis. TUNEL (green) and DAPI (blue) immunofluorescence staining of mouse choroid/RPE/retina cryosections from the normal: PBS and 0.5% PFD groups (IPL: inner plexiform layer; INL: inner nuclear layer; OPL: outer plexiform layer; ONL: outer nuclear layer; IS: inner segment; OS: outer segment). Scale bar: 200 *μ*m.

The cytotoxicity of 0.5% PFD was further evaluated by TUNEL. One day after the injection of PBS and 0.5% PFD in untreated mice, TUNEL staining indicated that normal, PBS and 0.5% PFD-injected groups showed very few TUNEL + cells in the retina after injection (Figure 4). Apoptotic cells in PBS and 0.5% PFD-injected groups were not significantly different from the normal group (Figure 4). In conclusion, 0.5% PFD injection is nothing more cytotoxic to the retina than PBS injection.

## 4. Discussion

The CNV mouse model is widely recognized and has been widely adopted to investigate wAMD mechanisms and to develop therapeutic strategies. Our current study was primarily focused on the effect of PFD in choroidal neovascular fibrosis and the possible underlying signaling pathway. In the present study, we first demonstrated that PFD significantly hindered choroidal neovascular fibrosis by using the CNV mouse model. The areas of choroidal fibrosis were dramatically decreased after PFD treatment (Figure 3), indicating that PFD has the potential effect of suppressing Col-I. Moreover, Western blot analysis showed that the overexpression of TGF-*β*2 significantly induced the phosphorylation of Smad 2/3, which directly activated the accumulation of*α*-SMA progression (Figure 2). But PFD markedly inhibited the related proteins expression of TGF-*β*/Smad signaling pathway in CNV. Furthermore, we demonstrated the antifibrotic efficacy of PFD in the CNV mouse model without causing intraocular toxicity, as verified by an apoptosis assay (Figure 4). These findings indicated that PFD was a potential inhibitor of subsequent subretinal fibrosis.

PFD is an oral pyridine drug, which can regulate various cytokines, change the synthesis and accumulation of collagen expression, inhibit the proliferation and expression of ECM, and has the effects of anti-inflammation, antioxidation, and antifibrosis [19]. It has been approved for clinical treatment of idiopathic pulmonary fibrosis [20], cirrhosis [21], and diabetic nephropathy [22], but its specific mechanism has not been fully elucidated. So far, the mechanism of PFD as fibrosis inhibitor in CNV has not been elucidated. TGF-*β* is a multifunctional cytokine regulating morphogenesis, proliferation, metabolism, apoptosis, and repair [23]. TGF-*β* is stored in the extracellular matrix and regulates the vascular matrix, leading to cell changes and angiogenesis [24]. Changes in the expression of TGF-*β* and its receptors in the vitreous, retina, and retinal pigment epithelium are closely related to CNV associated with retinal fibrosis and wAMD in proliferative vitreoretinopathy [25]. Studies have shown that TGF-*β*1 and TGF-*β*2 are upregulated during the formation of experimental subretinal fibrosis and are the major expression subtypes in the posterior segment of the eye [26]. Luo et al. Reported that pirfenidone could cause a reduction in TGF-*β*2 mRNA levels and mature TGF-*β*2 protein levels, which is due to reduced expression and direct inhibition of the TGF-*β* proprotein convertase furin [27]. In ARPE-19 cells, TGF-*β*2 promoted cell invasion into a collagen gel and induced the expression of collagen type I and fibronectin [28–30], two of the most prominent ECM components in subretinal fibrosis that play important roles in cell migration [31]. Therefore, before the study began, we finally selected pirfenidone by consulting the literature and reagents. Because tissue fibrogenesis is a complex process that has several cascades, involving inflammatory cell infiltration, apoptotic cell death, ECM expansion, and fibroblast activation, we analyzed the expression of different biomarkers that are widely used as sensitive markers for fibrosis, such as TGF-*β*2, type I collagen, and *α*-SMA. The TGF-*β* signal transduction pathway plays an important role in fibrosis of various organs and neovascularization, but its role in subretinal neovascular fibrosis is unclear. So we used a CNV mouse model to study the relationship between TGF-*β* signal transduction and CNV fibrosis. We observed that laser injury significantly increased the expression of TGF-*β*/Smad signaling from 1 week early to 4 weeks. After PFD treatment, the area of choroidal fibrosis was significantly decreased, indicating that PFD has the potential to inhibit fibrosis. Therefore, we investigated whether this effect is linked to the inhibition of the TGF-*β*/Smad signaling pathway. The use of the TGF-*β* inhibitor PFD before fibrosis formation can significantly reduce the extent of fibrosis areas. PFD also directly suppressed the deposition of ECM proteins, such as *α*-SMA, by inhibiting the activation of the TGF-*β*/Smad signaling pathway. Specifically, it may be involved in regulating the level of phosphorylation at different sites of Smad2/3, and further studies are required. In addition, PFD alleviated fibrosis formation without causing intraocular toxicity, as verified by an apoptosis assay. The above results indicate that PFD can reduce the degree of choroidal neovascular fibrosis in mice.

In summary, our data indicated that these effects of PFD were at least partly mediated via inhibition of the TGF-*β*/Smad signaling pathway. PFD can significantly inhibit the expression of TGF-*β*2 in the later stage of CNV, thereby weakening the Smad signaling pathway and further delaying the formation of the occurrence of subretinal fibrosis. Therefore, PFD may be a new and effective adjuvant drug for the treatment of wAMD. In the future study, it is important to determine how PFD exerts its effects on TGF-*β*2 and to confirm whether the agent can be used therapeutically or prophylactically for wAMD. These limitations will provide directions for our further research.

In conclusion, our studies suggest that PFD may be an effective therapy for choroidal neovascular fibrosis. Selective blockade of the TGF-*β*/Smad signaling pathway may be a mechanism by which PFD ameliorates subretinal fibrosis. This suggests that PFD might be a promising therapeutic target for the treatment of wAMD in clinical practice.

## Data Availability

The datasets used and/or analyzed during the current study are available from the corresponding author upon reasonable request.

## Abbreviations

TGF-*β*: Transforming growth factor-*β*
PFD: Pirfenidone
CNV: Choroidal neovascularization
PBS: Phosphate-buffered saline
OCT: Optical coherence tomography
FFA: Fundus fluorescein angiography
HE: Hematoxylin-eosin
CD: Cervical dislocation
Col-I: Collagen type I
p-Smad 2/3: Phosphorylated Smad 2/3
*α*-SMA: *α*-smooth muscle actin
TUNEL: Terminal deoxynucleotidy1 transferase dUTP nick-end labelling
wAMD: Wet age-related macular degeneration
RPE: Retinal pigment epithelium
ECM: Extracellular matrix
TGF*β*BR: TGF-*β* receptor
CTGF: Connective tissue growth factor
RT: Temperature
PFA: Paraformaldehyde
SDS–PAGE: Sulfate–polyacrylamide gel electrophoresis
PVDF: Polyvinylidene difluoride
HRP: Horseradish peroxidase
TBS: Tris-buffered saline
SD: Standard deviation.
